# *Himatanthus bracteatus* stem bark ethanolic extract obtained by sequential pressurized liquid extraction: Chromatographic characterization and profiling of cytotoxic, antitumoral and immunopharmacological properties

**DOI:** 10.1016/j.jtcme.2024.06.004

**Published:** 2024-06-12

**Authors:** Rose N. Pereira-Filho, Wilson D. Gonçalves-Júnior, Agenor G. dos Santos-Neto, John L.S. Cunha, Oslei P. de Almeida, Luciana N. Andrade, Daniela Droppa-Almeida, Ricardo G. Amaral, Cláudio Dariva, Juliana C. Cardoso, Patricia Severino, Eliana B. Souto, Ricardo L.C. de Albuquerque-Júnior

**Affiliations:** a Research and Technology Institute, Tiradentes University, Aracaju, 49010-390, Brazil; b Post-Graduating Program in Biotechnology, Brazilian Biotechnology Northeast Network (Renorbio), Tiradentes University, Aracaju, 49010-390, Brazil; c Post-Graduating Program in Health and Environment, Tiradentes University, Aracaju, 49032-490, Brazil; d Center of Biological and Health Sciences, Federal University of Western Bahia (UFOB), Barreiras, Bahia, 47810-047, Brazil; e Post-Graduating Program in Stomatopathology, University of Campinas, Campinas, São Paulo, 13083-970, Brazil; f Department of Medicine, Federal University of Sergipe, Lagarto, 49400-000, Brazil; g Department of Physiology, Federal University of Sergipe, São Cristóvão, 49100-000, Brazil; h Post-Graduating Program in Process Engineering, Tiradentes University, Aracaju, 49010-390, Brazil; i Laboratory of Pharmaceutical Technology, Department of Drug Sciences, Faculty of Pharmacy, University of Porto, 4050-313, Porto, Portugal; j Department of Pathology, Federal University of Santa Catarina, Florianópolis, Santa Catarina, 88040-370, Brazil

**Keywords:** Sequential pressurized *Himatanthus bracteatus* extract, Chromatographic analysis, Antitumor drug screening assays, Apoptosis

## Abstract

This study aims to characterize the cytotoxic, antitumoral and immunopharmacological profile of the ethanolic extract of *Himatanthus bracteatus* (EEHB) stem bark. Chromatographic analysis revealed the major EEHB composition in dimethyl isoplumerideo acid, 13-deoxyplumerido, isoplumeride, and plumeride. Cytotoxicity was performed on MCF-7 and MCF-10A cell lines using MTT assay. The antitumor activity was assessed using sarcoma 180 tumor cells subcutaneously implanted in mice. After seven days, hematological and biochemical analysis, and pathological evaluation of tumors and visceral organs were carried out. The IC_50_ value was 28.49 ± 2.05 μg/mL on MCF7 cells, but over 320 μg/mL on MCF-10A cells. Molecular docking was predicted using the caspase 3 molecular target with plumeride and isoplumeride ligands. Both compounds were also analyzed by PreADMET. The tumor growth inhibition was comparable to 5-FU. EEHB reduced the proliferative index (Ki67 immunoexpression) but increased the expression of apoptotic markers in a sarcoma 180 model. The ligands showed interaction with Caspase 3 with a binding energy between −7.2 and −6.6 kcal/mol for isoplumeride and −7.8 to −7.0 kcal/mol for plumeride. Hydrogen interactions were present between the ligands and caspase 3. Both phytochemicals showed low or no permeability in blood-brain barrier and medium permeability in Caco-2 cells and only isoplumeride showed mutagenic potential and carcinogenic. EEHB presented no toxicological effect either on the hematological parameters or average weight and histological features of liver, kidneys, and spleen. Our data suggest that EEHB has antitumor activity in S-180 tumor-bearing mice by blocking cell cycle and increasing apoptosis.

## Graphical abstract

**Figure:**
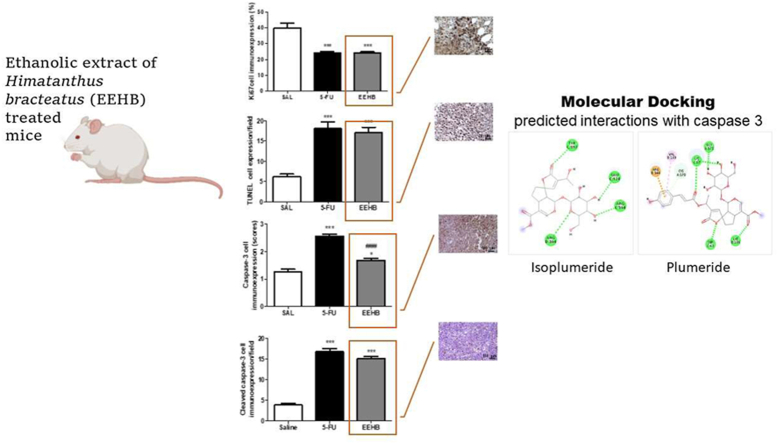

## 1 Introduction

Cancer is a group of diseases characterized by the loss of control and regulation of cell division and differentiation, leading to the continuous and autonomous proliferation of cells that invade tissues and organs.^1^ The pathogenesis of cancer encompasses multifactorial genetic alterations resulting from interactions between a range of risk factors (*e.g.,* the use of pesticides, tobacco, alcohol, exposure to ultraviolet radiation), promoting cellular, molecular and metabolic changes that favor the development of the disease.^2^ It is estimated that the number of new cancer occurrences in the world will reach 21.7 million new cases and 8.2 million cancer deaths worldwide by 2030.^3^

A multidisciplinary approach is commonly used to treat cancer, involving chemotherapy, radiotherapy, and surgical excision.^4^ However, to date, antineoplastic chemotherapy has stood out among the treatment modalities used for the control and cure of these malignant neoplasms.^5^ Chemotherapy consists on the use of drugs that modify cell metabolism through mechanisms that block the growth of tumor cells, induce the death of tumor cells (apoptosis or necrosis) or promote a reduction in the tumor's blood supply (blocking angiogenesis), used either alone or combined.^6^^,^^7^ Despite technological and scientific advances in the search of new chemotherapeutics, this therapy has a range of adverse and side effects, such as vomiting, nausea, alopecia, anemia, mucositis, hemorrhagic cystitis, gastrointestinal disorders, immunosuppression,^8^, ^9^, ^10^ thromboembolisms,^11^ and infertility,^12^ which can substantially affect the quality of life of patients.

Due to the toxic effects caused by cancer chemotherapy, many studies have turned to the search for new therapeutic approaches, more selective and with fewer side and adverse effects, based on the identification of compounds capable of replacing current drugs or allowing for a reduction in dosage of conventional chemotherapy.^13^, ^14^, ^15^ Thus, *in vivo* models that reproduce the natural history of human cancers and their clinical response to antineoplastic therapy have been used for the development of these studies, particularly those using sarcoma 180 (S180).^16^^,^^17^

The use of plants for therapeutic purposes has been a traditional habit in various cultures and has been considered a possible complementary therapeutic approach to combat cancer.^18^^,^^19^ Several studies involving the genus *Himatanthus* have been published, demonstrating its efficiency in *in vitro* and *in vivo* antitumor assays.^20^^,^^21^

Extracts obtained from plants of this genus are rich in iridoid compounds, such as plumeridoids,^22^ plumericin and isoplumericin,^23^ whose *in vitro* antitumor potential has been previously demonstrated.^24^ Regarding the *H. bracteatus* species, there are only reports of chemical composition studies,^25^ and no *in vitro* or *in vivo* assessment of a possible antitumor effect has been reported so far.

The different extraction methods and solvents used to extract phytoconstituents significantly vary the content of secondary metabolites obtained, which can influence the final composition of the extract as well as its biological properties.^26^ Sequential extraction is a technological strategy used to improve the extraction efficiency and biological activity of plant extracts, which consists of treating the sample with a “sequence” of different solvents of increasing or decreasing polarity, depending on the target substance to be obtained (if of high or low polarity, respectively). This method can be used not only for obtaining but also for the purification and concentration of chemical compounds.^27^ In addition, Pressurized Liquid Extraction (PLE) is a clean (green) extraction technique, performed under high-pressure conditions, that reduces the volume of solvents needed for extraction due to increasing the desorption kinetics of bioactive from the matrices, minimizing the use of organic solvents during extractive procedures.^28^ The extraction process using pressurized liquids also provides a shorter extraction time, and a more selective extraction of bioactive compounds, which reduces costs in the industrial process of purification of substances.^29^

Considering the pharmacological potential of the Apocynaceae family and the limited number of studies about *H. bracteatus* species, the purpose of this work is to assess the cytotoxic and antitumor effect of the ethanol extract of the stem bark of *Himatanthus bracteatus* obtained by sequential extraction under pressurized conditions.

## 2 Material and methods

### 2.1 Production of ethanol extract of *H. bracteatus* (EEHB) using sequential extraction with increasing polarity solvents in pressurized liquids

The bark of *H. bracteatus* was collected in February 2019, in the “Cipoal”, a community of the city of Óbidos, Pará State, Brazil [S 01° 44′ 32″ W 55° 28′ 13″ W] in winter (rainy season) in the Amazon region. A voucher specimen (number ASE-26.808) was deposited at the Herbarium ASE of the Federal University of Sergipe (ASE/UFS). The bark was dried (room temperature, for one week) and crushed (0.60–0.85 mm) for further extraction. The sequential extraction was carried out using the same experimental unit reported by Rodrigues Barbosa et al. (2020).^30^ Approximately 10 g of the bark of *H. bracteatus* were subjected to pressure liquid extraction (PLE, 25 °C, 100 bar, 1 mL/min for 25 min) for each solvent with increasing distinct polarity (hexane, dichloromethane, ethyl acetate, and ethanol). Between each solvent exchange, the system was depressurization and CO_2_ (30-0 bar). The material extracted using the first three solvents was discarded and the last ethanol extract was collected for further analysis. The residual solvent of the extraction was evaporated in a hot-air circulation oven at 40 °C. All experimental conditions were carried out at least in triplicate. The extraction yield was calculated by the relation of final weight (dry mass after extraction) and initial weight (bark powder) and expressed as a percentage.

### 2.2 Chemical characterization of the ethanol extract of *H. bracteatus* (EEHB) using ultra-efficiency liquid chromatography coupled to the diode array detector and mass spectrometry (CLUE-DAD-EM)

The EEHB (10 g) was solubilized in a solution of water: methanol (20 %) and centrifuged for 10 min. Subsequently,1 mg/mL of the material was injected and analyzed in an ultra-efficiency liquid chromatography system (Shimadzu) coupled with a high-resolution mass spectrometer (Compact Bruker Q Tof HRMS). Data analysis was performed in Compass 4.2 (ESI analysis) in negative mode, with 4500 capillary voltage, 500 V endplate, charging voltage 2000V, nebulizer at 5.5 bar, dry heater at 220 °C, dry gas at 12.0 L/min, and mass of *m*/*z* 50–1500 for MS. Separation was performed on a Shimpack ODS3 column (100 × 2.0 mm, 2.1 mm). The flow was 0.3 ml/min and the solvent system was 0.1 % formic acid (phase A) and acetonitrile (phase B) in a dilution gradient of 3 %–70 % of phase A for 16 min.

### 2.3 Cell cytotoxicity assay

The cytotoxicity of the EEHB was assessed in the human breast cancer cell line (MCF-7) and non-tumorigenic epithelial cell line (MCF-10A), acquired from the Rio de Janeiro cell bank (Brazil). The cells were grown in cell culture bottles (75 cm^3^, 250 mL volume), with RPMI 1640, supplemented with 10 % fetal bovine serum and gentamicin at 20 μg/ml, and kept under controlled conditions (5 % CO_2_ at 37 °C). Cell growth was monitored daily using an inversion microscope, and the medium was changed whenever the cell growth achieved the confluence necessary for nutrient renewal. Cells were plated in 96-well plates (1 × 10^6^ cells/mL, 100 μL/well) for cell viability tests using the 3-(4,5-dimethyl-2-thiazol)-2,5-diphenyl-2-H-tetrazolium bromide (MTT) salt method. After 24 h, EEHB was dissolved in 0.3 % dimethyl sulfoxide (DMSO) and added to each well in e concentrations of 10, 20, 40, 80, 160, and 320 μg/mL (100 μL). The experiment was carried out in three independent moments in triplicate, using 0.25 μg/mL doxorubicin and 0.3 % DMSO as a positive and negative control, respectively. The plates were incubated for 72 h in an oven with 5 % CO_2_, at 37 °C and the 96-well plate was read in a spectrophotometer (MULTISKAN-EX) at a wavelength of 570 nm. The percentage of cell viability was calculated as follows:

$$CV=\left[\frac{T}{NC}\right]\times 100$$

where T stands for EEHB absorbance and NC stands for absorbance of the negative control.

### 2.4 Antitumor assay in mice

Thirty male Swiss mice (20 ± 2 g) were housed in cages with free access to food and water, under controlled temperature (25 ± 2 °C) and relative humidity (50 ± 5 %), with a 12 h:12 h light-dark cycle (lights on at 6:00 a.m.). The ethical principles determined by the National Animal Experiment Control Council (CONCEA-Brazil) for experiments in animals were applied in this study, which was approved by the Ethical Committee for Animal Experimentation (approval nº 031115) and carried out according to the current guidelines for the care of laboratory animals. The *in vivo* antitumor study was performed as previously reported.^31^ Briefly, 10-day-old sarcoma 180 ascites tumor cells (2 × 10^6^ cells per 500 μL) were implanted subcutaneously into the left hind groin of the mice. The mice were assigned into five groups (n = 10) as shown in Table 1.

**Table 1:** Distribution of the animals in the experimental groups according to the treatment.

Groups (n = 10)	Transplantation procedures^a^	Treatment (i.p)
**SAL**	2 × 10^6^ cells/0.5 mL/mice	Saline solution (1 mL)
**5-FU**	2 × 10^6^ cells/0.5 mL/mice	5 FU (25 mg/kg)
**EEHB**	2 × 10^6^ cells/0.5 mL/mice	Ethanol extract of *H. bracteatus* (30 mg/Kg)
**Healthy animals**		

a 10-day-old sarcoma 180 cells (2 × 106 cells per 500 μL) obtained from ascitic tumor implanted subcutaneously into the left hind groin of the mice.

One day after implantation, the EEHB was suspended in saline (vehicle) at a final concentration of 30 mg/mL and administered intraperitoneally daily for seven days. Saline solution (1 mL) was used as a negative control, whereas 5-fluorouracil (5-FU, purity >99 %; Sigma Chemical Co., 25 mg/kg) was used as a positive control. On day 8, peripheral blood samples were collected from the orbital plexus of the mice, under light ether anesthesia, for further hematological analysis. Subsequently, the mice were euthanized using a lethal anesthetic dose (0.5 mL of 2 % xylazine/100 g and 0.5 mL of 10 % ketamine/100 g). The tumors were excised, weighed, and histologically processed. Then, the specimens were formalin-fixed and paraffin-embedded for histological analysis. The relative tumor growth inhibition (TGI) was calculated by the following equation:

$$TGI\left(\%\right)=\left[\frac{A-B}{A}\right]\times 100$$

where A stands for the mean tumor weight in the negative control group, and B for average tumor weight in the treated animals.

### 2.5 Pathological analysis of tumors

Histological sections (5 μm thick) stained in Hematoxylin and Eosin (HE) were used for pathological examinations of the tumors, considering the intensity of cytological atypia, extent of coagulative necrosis, and the number of mitotic figures, categorized into four scores, as shown in Table 2. The presence of muscle, adipose and perineural invasion, as well as tumor emboli formation, was also evaluated.

**Table 2:** Effect of the ethanol extract *of Himatanthus bractetus* on the hematological parameters of blood collected via the orbital plexus in animals transplanted with Sarcoma 180 tumor cells.

Hematological parameter	Treatment (*i.p.*)			Healthy animals
	Saline	5-FU (25 mg/kg)	EEHB (30 mg/kg)	
**Red cells (10**^**6**^ **cells/mL)**	7.5 ± 1,1^a^	7.1 ± 1.8^a^	8.3 ± 1.6^a^	6.9 ± 1.3^a^
**Hematocrit (%)**	41.4 ± 1.8^a^	44.0 ± 1.1^a^	40.9 ± 2.3^a^	42.3 ± 2.1^a^
**Hemoglobin (g/dL)**	15.2 ± 1.3^a^	12.0 ± 2.6^a^	12.2 ± 2.3^a^	14.1 ± 1.5^a^
**Total leukocytes (10**^**3**^ **cellsμL)**	30.6 ± 9.3^a^	6.6 ± 3.2^b^	39.0 ± 17.2^a^	10.0 ± 1.2^c^
**Segmented Neutrophils (%)**	61.5 ± 1.1^a^	10.5 ± 6.5^b^	55.4 ± 5.9^a^	31.3 ± 4.4^c^
**Band Neutrophils (%)**	2.0 ± 0.7^a^	0.0 ± 0.0^b^	2.3 ± 0.4^a^	2.1 ± 0.5^a^
**Monocytes (%)**	2.3 ± 0.8^a^	1.0 ± 1.6^a^	1.6 ± 0.5^a^	1.0 ± 0.1^a^
**Lymphocytes (%)**	33.8 ± 1.1^a^	87.6 ± 14.8^b^	60.4 ± 5.5^b^	68.9 ± 1.04^b^

Data are expressed as Mean ± Standard Deviation. Significant differences between values (p < 0.05) are expressed as different letters in the same line (ANOVA followed by Tukey's test).

### 2.6 Assessment of *in situ* DNA fragmentation using terminal deoxyuridine nick-end labeling (TUNEL) staining

The number of apoptotic cells was assessed by the TUNEL technique using an *in situ* cell death detection kit, POD (Roche Diagnostics, Indianapolis, IN, USA), as described by.^32^ Histological sections (5 μm thick, n = 5) were deparaffinized in xylene, rehydrated in alcohol (99, 95, and 70 %), and washed with deionized water. Then, the samples were treated with proteinase K (20 μl/ml in PBS), and endogenous peroxidase activity was quenched with 2 % H_2_O_2_ (10 min at room temperature). and washed with PBS buffer. The sections were incubated with TdT enzyme solution (37 °C, 1 h) and washed again with PBS buffer. The sections were incubated with anti-digoxigenin peroxidase (50 μL, 30 min), and washed with PBS once again. Diaminobenzidine (DAB, Ventana Medical Systems, Tucson, AZ, USA) was used to reveal the reaction. For negative controls, the TdT enzyme was replaced with PBS on one section on each slide and was processed in parallel. Counterstaining of nuclei was performed with 2 % Meyer's hematoxylin and mounted for examination. TUNEL-positive cells were identified by their brown-stained nuclei or as apoptotic bodies (fragments of apoptotic cells engulfed by neighboring cells). The relative number of TUNEL-positive cells was calculated according to the following equation:

$$TPc\left(\%\right)=\left[\frac{Tn}{1000}\right]\times 100$$

where TPc (%) is the relative of TUNEL-positive cells (percentage) and Tn is the absolute number of TUNEL-positive cells in the sample.

### 2.7 Immunohistochemical analysis of expression of Ki67 cell proliferation antigen and pro-apoptotic enzymes caspase-3 (wild-type) and cleaved caspase-3

Histological sections (3 μm thick, n = 5) were mounted on glass slides previously silanized and subjected to immunohistochemical reaction through the indirect streptoavidin-biotin method. Sections were deparaffinized in xylene and washed in decreasing concentrations of ethyl alcohol (100 %, 95 %, 90 %, 80 %, and 70 %). Blocking endogenous peroxidase was performed with hydrogen peroxide at 3 % and methyl alcohol (10 min in a darkroom). The antibodies anti-caspase-3 and anti-active + pro-caspase-3 (Sigma, São Paulo, Brazil) were incubated for 30 min. The reaction was revealed using diaminobenzidine (DAB, Ventana Medical Systems, Tucson, AZ, USA) and counterstained with Meyer's Hematoxylin. Both steps were developed at an interval of 4 min each.

Positive control was performed with a sample of high-grade malignant neoplasm (lymphoma). For the negative, the primary antibody was replaced by buffered saline solution with phosphate in the reaction. The intensity of Caspase-3 immunopositivity was determined using a histological grading system: 0 (lack of positive cells), 1 (<30 % of positive cells), 2 (30 %–60 % positive cells) 3 (>60 % of positive cells). The mean number of Ki67 and Cleaved Caspase-3 immunopositive cells was calculated according to the following equation:

$$IPc\left(\%\right)=\left[\frac{PCn}{1000}\right]\times 100$$

Where IPc(%) is the relative number of Ki67 or Cleaved caspase-3 immunopositive cells (percentage) and PCn is the absolute number of immunopositive cells in the sample.

### 2.8 Assessment of the potential toxicological effects

The analysis of the toxicological effects of EEHB was assessed in the experimental groups (SAL, 5-Fu, and EEHB), and in an additional group of 10 healthy mice. Briefly, the body weight and food/water intake of the animals were recorded on days 0, 2, 4, 6, and 8. Regarding the hematological analysis, leukocyte (total and differential leukocytes) and erythrocyte (red blood cells, hemoglobin, and hematocrit) parameters were evaluated. The total leukocyte count was assessed with 20 μL of blood, diluted in 380 μL of Turk. Then, 10 μL of the suspension was placed in a Newbauer chamber and total leukocytes were counted. The differential leukocyte count was determined using a drop of whole blood added to a clean slide and identified for the preparation of blood smears, stained with panoptic dyes (Pinhais, Brazil, Instant-Prov®). Leukocyte cells (basophils, eosinophils, lymphocytes, monocytes, and neutrophils) were identified using an optical microscope. Blood samples collected in a heparinized cannula were placed in a tube containing the EDTA anticoagulant for the evaluation of erythrocyte parameters (red blood cells, hemoglobin, and hematocrit) in an automated analyzer (BC5380, Mindray, Shenzhen, China). After euthanasia (previously described) of the animals, the spleen, liver, and kidneys were excised, and their relative weight (g/100 g of body weight) was determined. Subsequently, the organs were examined regarding gross morphology, formalin-fixed and paraffin-embedded. Hematoxylin/eosin-stained histological sections (5 μm thick) were used for pathological analysis.

### 2.9 Molecular docking of the EEHB major phytochemicals

The major phytochemicals were subjected to molecular docking with the molecular target Caspase-3 (PDB: 3DEI) is an attractive therapeutic target for the treatment of diseases involving dysregulated apoptosis. The structures were obtained free of charge from the Protein Data Bank (PDB) (https://www.rcsb.org) as a theoretical 3D model. The 3D structures of the phytochemicals were downloaded from the PubChem database (http://pubchem.ncbi.nlm.nih.gov). Receptor and ligand residues were prepared and molecular docking simulations were performed using the AutoDock Vina 4 program, a software widely used due to the high accuracy of predictions and their speed.^33^^,^^34^ Water molecules and cofactors were removed and polar hydrogens were added to the protein to see the protein-ligand interaction.^35^ The free version of the Biova Discovery Studio 2020 client software was used to visualize the interactions.^36^ Only plumeride and isoplumeride were tested.

### 2.10 *ADMET (absorption, distribution, metabolism, excretion, and toxicity)* analysis of the EEHB major phytochemicals

The ADMET was the offline computational tool used to evaluate the profile of the major phytochemicals found in the EEHB regarding the characteristics of the compounds, pharmacokinetics, mutagenicity, and toxicological dose level for different tissues. Pharmacologically relevant properties were predicted using the PreADMET server (http://preadmet.bmdrc.org/).

### 2.11 Statistical analysis of data

Data expressed as mean ± SEM (standard error of the mean) were subjected to the Shapiro-Wilk normality distribution test. Data with normal distribution were compared using ANOVA followed by multiple comparisons Tukey's test. Data with non-normal distribution were compared using the Kruskal-Wallis test with multiple comparisons Dunn's test. Differences between means were considered significant when p values were less than 0.05.

## 3 Results

### 3.1 Yield and chemical characterization of the EEHB

The extraction yield was 2.31 ± 0.64 % Chromatographic analysis revealed four major chemical compounds in the extract: dimethyl isoplumerideo acid, 13-deoxyplumerido, isoplumeride, and plumeride (Supplementary material S1).

### 3.2 EEHB exerted *in vitro* and *in vivo* antitumor effect comparable to 5-fluorouracil (5-FU)

**Fig. 1:**
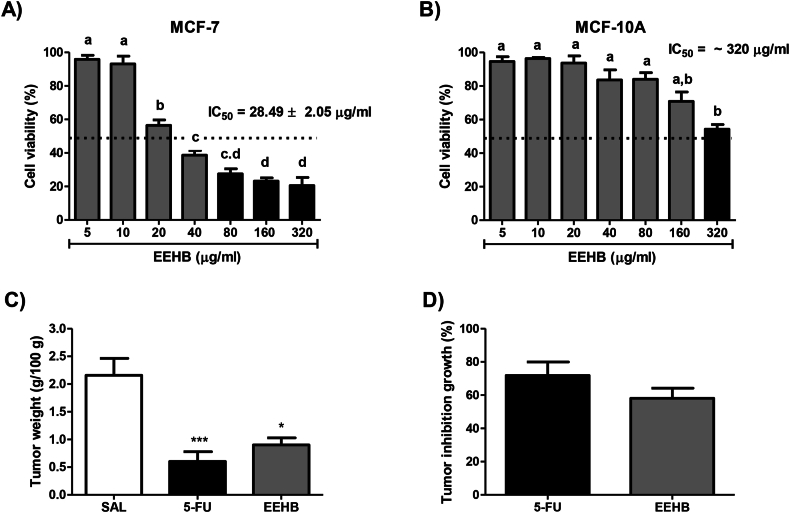
Assessment of the cytotoxicity of the sequential ethanol extract of *Himatanthus bracteatus* (EEHB) on MCF7 (A) and MCF10A (B) cells using increasing molar concentration (determined based on the content of plumericin and isoplumericin). (C) Assessment of mean weight of experimental tumors in mice induced by implantation of sarcoma 180 cells and (D) mean percentage of tumor growth inhibition in relation to the mean tumor weight of the group treated only with saline solution. The groups were treated with saline solution (SAL), 5-Fluorouracil (5-FU) and ethanol extract of *Himatanthus bracteatus* (EEHB). Data were presented as mean ± standard error mean. Significant differences in comparison with SAL were expressed as * p < 0.05 and ***p < 0.001 (ANOVA and Tukey's multiple comparisons test).

Fig. 1A/B shows the effect of the treatment with EEHB at increasing concentrations of MCF-7 cell line derived from mammary adenocarcinoma and MCF-10^a^ cell line derived from non-tumor mammary epithelial cells. EEHB showed moderate cytotoxic against MCF7 cells, with IC_50_ values of 28.49 ± 2.05 μg/ml, but quite lower cytotoxicity on MCF-10A cells (IC_50_ values over 320 μg/ml). Regarding the antitumor assay in the rodent model, tumors developed in 100 % of mice after seven days of tumor cells transplantation. As demonstrated in Fig. 1C/D, the mean tumor weight in both 5-FU (0.6 ± 0.1 g/100 g) and EEHB (0.9 ± 0.1 g/100 g) was significantly lower than in SAL (2.1 ± 0.3 g/100 g) (p < 0.001 and p < 0.05, respectively). Furthermore, no significant difference (p > 0.05) was found between the TGI observed in 5-FU (71.8 ± 8.1 %) and EEHB (58.1 ± 6.0 %).

### 3.3 EEHB reduced both the histological scores of coagulative necrosis and the mean number of mitoses in the sarcoma 180 model

**Fig. 2:**
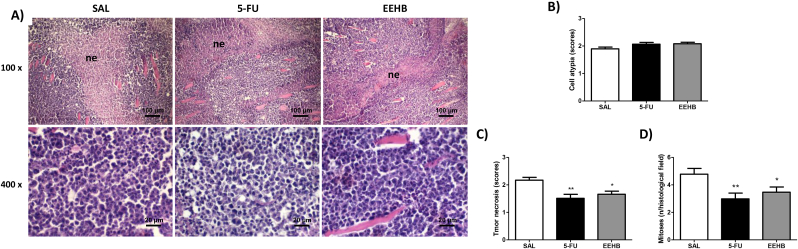
(A) Photomicrographs of HE-stained histological sections of sarcoma 180 tumors of the experimental groups showing the proliferation of sheets of round to polygonal-shaped cells with dark and moderately pleomorphic nuclei. Areas of coagulative necrosis are observed in the center of tumor sheets. (B) Assessment of the scores of cell atypia and (C) tumor necrosis, as well as (D) the mean number of mitoses/histological field (p.00065 mm^2^). Data are expressed as mean ± standard error mean. Significant differences in comparison with SAL are expressed as *p < 0.05 and **p < 0.01 (Kruskal Wallis and Dunn's multiple comparisons test).

Pathological analysis of the tumors showed intense and diffuse proliferation of polygonal cells, presenting large eosinophilic cytoplasm and bulky nuclei, with variable degrees of atypia. Tumors often exhibited central areas of coagulative necrosis surrounded by lymphocytic and polymorphonuclear infiltrate (Fig. 2A). Tumor cells infiltrated the skeletal striated muscle tissue, hypodermic adipose panniculus, peripheral nerves sheath, and mammary ducts. Moreover, the areas of necrosis were predominantly seen as thin irregular bands or trabeculae in the center of the tumors in 5-FU and EEHB, but as large clumps of variable diameter in SAL. As shown in Fig. 2B/C/D, there was no significant difference in the cytological atypia scores between SAL (1.9 ± 0.1), 5-FU (2.0 ± 0.1) and EEHB (2.0 ± 0.1) (p > 0.05). However, the content of coagulative necrosis and number of mitoses were significantly lower in 5-FU (1.5 ± 0.2 and 3.0 ± 0.4 mitosis/field; p < 0.01) and EEHB (1.6 ± 0.1 and e 3.6 ± 0.4 mitoses/field; p < 0.05) than in SAL (2.2 ± 0.1 and 4.1 ± 0.4 mitoses/field), but there was no significant difference between 5-FU and EEHB (p > 0.05).

### 3.4 EEHB promotes significant reduction in the proliferative index but increased the expression of apoptotic markers in a sarcoma 180 model

**Fig. 3:**
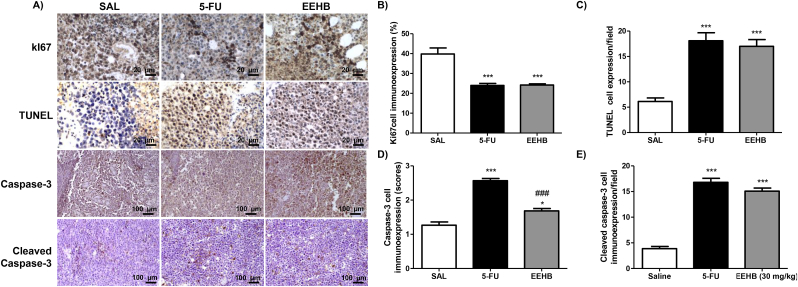
(A) Photomicrographs of histological sections of sarcoma 180 tumors of the experimental groups showing the nuclear pattern of expression. of Ki67 antigen and Terminal deoxynucleotidyl transferase (TdT) dUTP Nick-End Labeling (TUNEL) assay, as well as the cytoplasmic pattern of immunoexpression of the apoptosis-related enzymes Capase-3 and Cleaved Caspase-3 (LSAB method). (B) Quantitative analysis of tumor cells expressing the proliferation antigen Ki67 and the apoptosis markers (C) TUNEL, (D) Caspase-3 and (E) Cleaved Caspase-3. Data are expressed as mean ± standard error mean. Significant differences in comparison with SAL are expressed as *p < 0.05 and ***p < 0.001; significant differences in comparison with 5-FU are expressed as ^###^p < 0.01 (ANOVA and Tukey's multiple comparisons test).

As shown in Fig. 3, Ki67 and TUNEL positive cells were identified by brownish nuclear staining, whereas the immunoexpression pattern observed in caspase-3 and cleaved caspase-3 was cytoplasmic. The proliferative index (mean number of Ki67-positive cells) was significantly lower in 5-FU (23.9 ± 1.0) and EEHB (24.2 ± 0.5) in comparison with SAL (39.8 ± 3.1) (p < 0.001). The mean number of TUNEL-positive cells in 5-FU (18.0 ± 1.5 cells/field) and EEHB (17.0 ± 1.3 cells/field) was significantly greater in comparison with SAL (6.1 ± 0.6 cells/field) (p < 0.01). Similarly, the immunoexpression of apoptosis-related enzymes caspase-3 and cleaved caspase-3 was significantly increased in both 5-FU (2.5 ± 0.06 and 18,4 ± 0,7 cells/field) and EEHB (1.7 ± 0.06 and 16,4 ± 0,5 cells/field) compared with SAL (1.3 ± 0,09 and 3,8 ± 0,5 cells/field) (p < 0.001). Interestingly, the expression of caspase-3 was greater in 5-FU than in EEHB (p < 0.001), but no significant difference between them was observed regarding the expression of cleaved caspase-3 (p > 0.05).

### 3.5 EEHB presented no toxicological effect either on the hematological parameters or the average weight and histological features of the liver, kidneys, and spleen

As demonstrated in Table 2, no significant changes were observed in the red blood cell count between groups. Regarding the white blood cells count, while a significant increase in total leukocytes count, interpreted as leukocytosis, was observed in the groups treated with saline and EEHB (p < 0.01), a significant decrease in this hematological parameter was found in the 5-FU-treated group (p < 0.05) in comparison with healthy animals. The analysis of leukocyte differential counts demonstrated neutropenia related to the administration of 5-FU, both in mature and immature neutrophils (segmented and band granulocytes, respectively). A significant increase in lymphocytes was also observed in this group (p < 0.05), but it could not be considered as lymphocytosis because of the total leukopenia. Mild mature neutrophilia was observed in leukocytes differential counts EEHB-treated groups (p > 0.05). In addition, 5-FU, but not EEHB, promoted a significant decrease in the average weight of the spleen and kidneys (Table 3).

**Table 3:** Assessment of the effect of the ethanol extract of *Himatanthus bracteatus* (EEHB) on the relative organ weight of mice subjected to Sarcoma 180 tumor cells transplantation.

Treatment (i.p)	Dose (mg/kg/i.p.)	Liver (g/100 g)	Spleen (g/100 g)	Kidneys (g/100 g)
**Salina**	–	1.36 ± 0.11^a^	0.20 ± 0.04^a^	0.42 ± 0.02^a^
**5-FU**	25	1.29 ± 0.26^a^	0.08 ± 0.01^b^	0.28 ± 0.03^b^
**EEHB**	30	1.40 ± 0.08^a^	0.35 ± 0.04^a^	0.39 ± 0.07^a,b^
**Healthy animals**	–	1.43 ± 0.18^a^	0.37 ± 0.02^a^	0.40 ± 0.05^a^

Data are expressed as Mean ± Standard Deviation. Significant differences between values were expressed as different letters in the same column (ANOVA followed by Tukey's test).

**Fig. 4:**
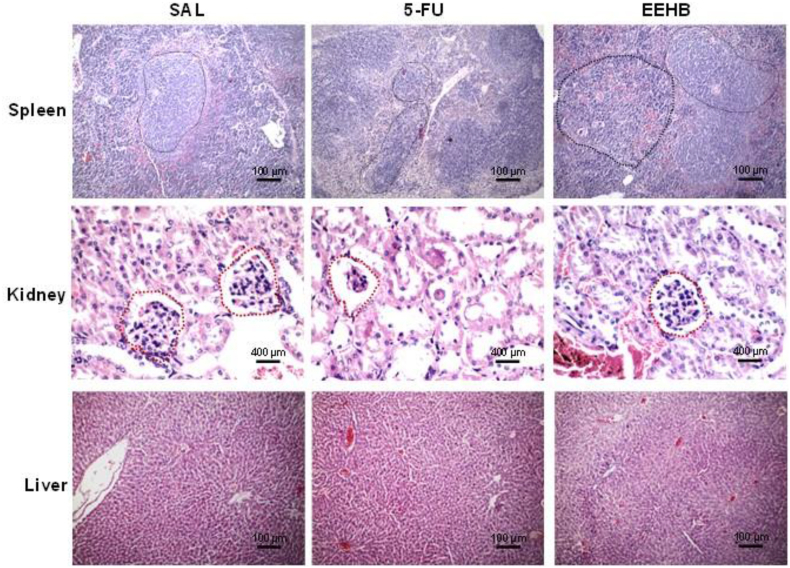
Photomicrographs of HE-stained histological sections of visceral organs of the experimental groups. 5-FU has a reduction in both splenic white pulp lymphoid follicles (100 × ) and renal glomeruli (400 × ) compared to saline and EEHB, but no pathological change is observed in the cytomorphological architecture of the liver samples (100 × ).

Those pathological effects were confirmed by histological analysis, which revealed atrophy of spleen white pulp and renal cortex, expressed as reduction of the lymphoid follicles and glomeruli diameters, respectively (Fig. 4). No pathological effects were observed in the animals' liver and kidneys, regardless of the group.

### 3.6 Molecular docking of plumeride and isoplumeride of EEHB

Both ligands were explicitly embedded in Caspase 3. According to the prediction by molecular docking, the binding affinity of isoplumeride with Caspase 3 ranged from −7.2 to −6.6 kcal/mol, whereas plumeride presented a variety of binding affinity −7.8 to −7.0 kcal/mol (Table 4).

**Table 4:** Binding affinity between Caspase 3 and isoplumeride and plumeride with their respective interactions at the active site.

Caspase 3	Affinity (kCal/mol)	Hydrogen bond
**Isoplumeride**	−7.2	GLU^124^ - 2.53 Å;
		ARG^164^ - 2.21 Å;
		TYR^197^ - 2.49 Å;
		ARG^164^ - 2.49 Å.
**Plumeride**	−7.8	LYS^186^ - 2.03 Å;
		LYS^57^ - 2.68 Å;
		LYS^57^ - 1.87 Å;
		LYS^57^ - 2.69 Å;
		SER^63^ - 2.04 Å;
		GLY^171^ - 2.59 Å.

**Fig. 5:**
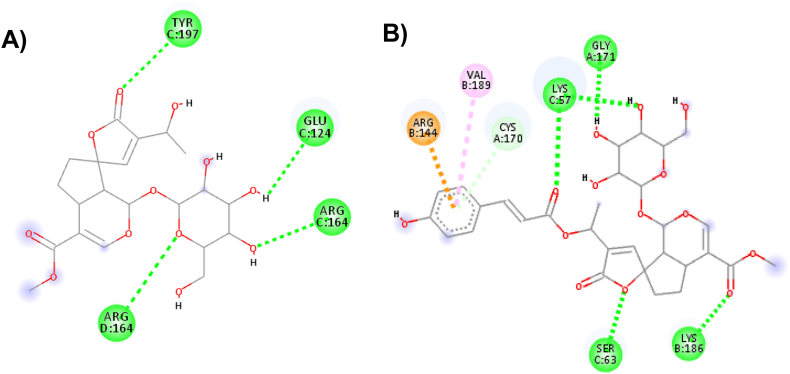
(A) 2D diagram with interactions predicted by molecular docking between isoplumeride and Caspase 3. (B) 2D diagram with interactions predicted by molecular docking between plumeride and Caspase 3.

The main types of intermolecular interactions observed were hydrogen bonds and covalent bonds. Fig. 5A presents the amino acids responsible for the interaction between Caspase 3 and the isoplumeride, GLU^124^, ARG^164^ showing two interactions, TYR^197^ with a distance of 2.53 Å, 2.21 Å, 2.49 Å, and - 2.49 Å, respectively. This type of interaction occurs when hydrogen is attracted by a more electronegative atom, which in this case was oxygen present in the carboxylic group of the amino acid GLU^124^, while with the other amino acids the interactions occurred through the hydrogens from the side chain. The interactions type hydrogen, electrostatic and hydrophobic bonds of plumeride with caspase 3 are demonstrated in Fig. 5B. The amino acids involved in these hydrogen bond-like interactions are LYS^186^, LYS^57^ at three points, SER^63^ and GLY^171^ with a distance of 2.03 Å, 2.68 Å, 1.87 Å, 2.69 Å, 2.04 Å, and 2.59 Å, respectively. LYS and the other amino acids had their interactions with plumeride through the side chain and oxygen present in plumeride. The hydrophobic and electrostatic bond was provided by the presence of an aromatic ring in the chemical structure of the plumeride. The hydrophobic bond was Pi-Alkyl type with VAL^189^ at a distance of 5.31 Å, whereas the electrostatic bond was Pi type-cation with the ARG^144^ at a distance of 3.55 Å.

### 3.7 ADMET profile of plumeride and isoplumeride of EEHB

The prediction and significant descriptors of the isoplumeride and plumeride are demonstrated in Table 5. Both phytochemicals showed low or no permeability with Blood-Brain Barrier (BBB) and medium permeability to Caco-2 cells (epithelial cell lines originating from human colon adenocarcinoma of the large intestine) and high skin permeability (<0.1). Regarding the metabolism, both phytochemicals showed inhibition scenarios for CYP450 components CYP2C19, CYP2C9, and CYP3A4, whereas they were substrates for CYP3A4 and CYP2D6. Although isoplumeride presented a higher MDCK value (15.8) compared to plumeride (0.04), both were considered compounds of low excretion via the MDCK pathway. Both compounds showed high solubility in water. Only plumeride inhibited the protein P-glycoprotein (PgP) whereas only isoplumeride was predicted as mutagenic potential and carcinogenic to mice.

**Table 5:** ADME properties obtained from the PreADMET server of the isoplumeride and plumeride compounds.

ADME prediction	Isoplumeride	Plumeride
*BBB	0.0239779	0.0245111
**Caco2**	18.0653	19.243
**Skin_Permeability**	−4.95066	−3.87432
*CYP_2C19_inhibition	Inhibitor	Inhibitor
**CYP_2C9_inhibition**	Inhibitor	Inhibitor
**CYP_2D6_inhibition**	Non	Non
**CYP_2D6_substrate**	Non	Non
**CYP_3A4_inhibition**	Inhibitor	Inhibitor
**CYP_3A4_substrate**	Weakly	Substrate
*MDCK	15.891	0.0461715
**Pgp_inhibition**	Non	Inhibitor
**Pure_water_solubility_mg_L**	soluble	soluble
**Toxicity Prediction**		
**Ames_test**	mutagen	non-mutagen
**Carcino_Mouse**	positive	positive
**Carcino_Rat**	positive	negative

## 4 Discussion

In this study, the cytotoxic effects of EEHB on malignant and non-malignant cell lines were assessed by MTT assay, as a screening strategy for the cytotoxic activity of the extract, to assess whether there was potential for a study using *in vivo* models, and not as an indication of induction of apoptosis. The IC_50_ value against the MCF-7 cell line (human breast carcinoma) was reported to be below 30 μg/mL, which is following the regulations defined by the United States National Cancer Institute program for anticancer drug screening for further development of anticancer drugs.^37^ Cytotoxic effect of approximately 20 % of cell growth inhibition promoted by methanol extract of the bark and leaves of *H. bracte*atus against HepG2 (human hepatocellular carcinoma) and HL-60 (human promyelocytic leukemia) cell lines at a concentration of 50 μg/mL has been previously reported.^38^ Therefore, the sequential extraction method used in the current study may have promoted a cleaning of the extract, and consequently a greater concentration of substances with anticancer activity, improving cytotoxicity properties and reducing the IC_50_ concentration of the extract. In addition, the 10 folds' greater IC_50_ value against the MCF-10A cell line (breast epithelial cells) suggests cytotoxic selectiveness against malignant cells. These data make the EEHB an eligible product for performing antitumor assays using *in vivo* models. Since the selective activity is suggested by the *in vitro* results, we performed the *in vivo* analysis to confirm the potential antitumor biological activity of the extract in a rodent model. Hence, the *in situ* increased DNA fragmentation and caspase-3 active overexpression, which was supported molecular docking and *in silico* results, brought the first data pointing to a possible activation of apoptosis as a mechanism to inhibit tumor growth *in vivo*.

The biological model chosen to carry out the study of antitumor activity was sarcoma 180. This experimental murine tumor can be developed by implantation of neoplastic cells in the dermal tissue of mice, and its main advantages are the ease of carrying out the tumor implantation protocol, tumor growth in 90–100 % of the animals, and rapid tumor evolution, reducing the time of experimentation (WEI, 2018). We found that EHHB inhibitory effect on sarcoma 180 growth was comparable to 5-FU. Although the mechanism underlying the EEHB antitumor activity has not yet been elucidated, it might be likely related to the presence of iridoids in its chemical composition. The antitumor potential of iridoids, major compounds of EEHB, has been well documented since the 80s.^39^ In addition, a comprehensive review of iridoids previously reported has shown that many of these molecules are capable of inhibiting tumor cell development,^40^ by altering the expression of Bax/Bcl-2, arresting cells in the cell cycle in the G2/M phase, thus preventing their migration and invasion. They are also involved in a signaling pathway present in numerous types of cancers, the pathway MAPK/ERK inhibiting the expression of important metalloproteases such as MMP-2 and MMP-9 inhibiting heterogeneous adhesion.^41^

Classic works published during the 80s confirm that some histopathological features of malignant tumors can be related to their biological behavior, such as mitotic activity, areas of necrosis, and cytological atypia.^42^ In this study, 5FU and EEHB had tumors with a lower mean mitotic index and fewer areas of necrosis. As the mitotic index has been associated with tumor proliferative activity and the formation of areas of necrosis is often associated with cell proliferation so intense and rapid that neovascularization does not occur quickly enough to provide nutrition to tumor cells^43^ those data are suggestive of a less aggressive biological behavior. However, as no significant difference was observed concerning cell atypia, none of the treatments seemed to influence the pattern of tumor differentiation.

The immunohistochemical expression of the Ki67 antigen is closely associated with the tumor growth fraction (number of tumor cells in the cell cycle), and this antigen is not expressed in the DNA repair phases or phase of proliferative rest.^44^ Therefore, the cell expression of ki67 was used in this study to determine whether the reduction in tumor weight *in vivo* was caused by a lower proliferative rate. Ki67 expression was significantly reduced in the EEHB-treated group similar to what occurred in the group treated with the chemotherapeutic agent. The mechanism of action of 5-FU is mainly related to the inappropriate incorporation of fluoronucleotides into DNA and RNA during their synthesis, as well as to the inhibition of the enzyme thymidylate synthase (TS), both occurring during the cell cycle. Thus, the 5-FU-induced lower immunoexpression of Ki67 in sarcoma 180 could be the result of both cell cycle blockage and apoptosis of cycling cells. However, hypothesizing a mechanism of action for the effect obtained with EHE 30 is more complex, particularly because this is the first study assessing the antitumor effect of EEHB *in vivo*. The antiproliferative effect of the ethanol extract of *Allamanda branchetti* and *Allamanda schottii*, rich in iridoids such as plumerides, on the growth of leukemic cells and non-malignant endothelial cells, has been previously demonstrated.^45^ Another study has demonstrated that plumericin isolated from *Momordica charantia* vine using the supercritical fluid extraction inhibited proliferation of NB4 and K562 leukemic cancer cell lines (effective doses of 4.35 ± 0.21 and 5.58 ± 0.35 μg/mL, respectively); however, the mechanism of growth inhibition has been associated with both G2/M arrest and apoptosis.^24^ Thus, the lower rate of EEHB-induced tumor growth could result not only from a supposed blockage of the cell cycle but also from an increase in the rate of apoptosis of neoplastic cells belonging to the proliferative tumor fraction.

The TUNEL *in situ* labeling method was used in the current study to assess the apoptotic index of tumor cells in response to EEHB treatment. Thus, the significant increase in TUNEL positive cells in the group treated with EEHB suggests that the antitumor effect of the extract may be related to the stimulation of apoptosis. However, classic works in the ‘90s have already shown that TUNEL can mark cells that have suffered severe DNA damage not associated with apoptosis, as occurs in some patterns of necrosis (such as in cases where nuclear fragmentation predominates or karyorrhexis) or even in autolytic cell death.^46^

Caspase-3 is a cysteine protease involved not only in the pathogenesis of apoptosis-related diseases but also in monitoring the effectiveness of chemotherapy.^47^ Therefore, it was assessed in the current study to determine whether the increase in the number of TUNEL-positive cells would be related to apoptosis or other forms of cell death. Overexpression of caspase-3 was found in both EEHB and 5-Fu-treated groups. As the immunohistochemical overexpression of caspase-3 in the experimental model of sarcoma 180 has been associated with the activation of the apoptotic cascade through the mitochondrial pathway,^48^ our data are suggestive of apoptosis-related antitumor activity played by EEHB. However, lack of caspase 7 expressions in cells expressing caspase-3 after stimulation of the extrinsic pathway of apoptosis has been demonstrated in the human carcinoma cell line.^49^ Despite being different biological models in many respects, this report suggests that the immunohistochemical expression of caspase-3, although more specific than TUNEL, is still not sufficient to reliably determine that cell death in a sarcoma 180 model occurred by the apoptotic route. Caspase-3 is cleaved and activated by caspase-8 and caspase-9 at an aspartate residue to produce a p12 and a p17 subunit that together form the active cleaved caspase-3 enzyme, the main executing caspase, responsible for cellular protein degradation and DNA fragmentation during apoptosis.^50^ Therefore, the overexpression of cleaved caspase-3 observed in the current study confirmed the EEHB-induced activation of the apoptotic cascade in the sarcoma 180 murine models. Interestingly, the immunoexpression of cleaved caspase-3 (active) was much less intense than the wild-type counterpart of the enzyme (uncleaved caspase-3), probably because the uncleaved forms of the enzyme are stable monomers in the cell (procaspases), that are converted into active protease by chain cleavage, acquiring high enzymatic activity only upon the induction of apoptosis signaling.^51^^,^^52^

Although the inhibition of caspase expression frequently occurs in cancer cells, making them “immortal” and resistant to chemotherapeutic agents,^53^ their overexpression may determine the release of growth-stimulating signals for non-apoptotic tumor cells, so that, in a sort of “rebound effect”, they proliferate and survive under stress conditions.^54^ Therefore, the overexpression of caspase-3 and cleaved caspase-3 is not enough to assure that the antitumor effect of EEHB is related to increased apoptosis. For this reason, the careful and combined analysis of data, including the cell proliferation rate, assessed by immunostaining for Ki-67, was necessary to provide evidence that the antitumor mechanism of EEHB is not only due to blocking the cell cycle but also to the activation of caspase-dependent apoptotic pathways.

Chemotherapy suppresses the hematopoietic system,^55^ and neutropenia is the most serious hematologic toxicity, which often limits the doses of chemotherapy that can be tolerated.^56^ In the current study, 5-FU induced strong immunohematological suppression, such as leukopenia and inversion of neutrophil/lymphocyte differential count, as well as reduction of relative spleen weight, likely related to white pulp atrophy, changes that have been also previously reported.^57^ However, no suppressive effect was observed in the animals treated with EEHB (30 mg/kg), either on both red and white cells of peripheral blood or in the weight and histological features of the spleen. Indeed, the EEHB promoted an increase in total leukocytes of peripheral blood, suggesting not only relative immunohematological safety in the use of this natural chemical compound but also a possible immunostimulatory effect. Previous studies have shown an ethanolic extract of *H. articulatus* bark induces immunomodulatory activity, such as stimulation of antibody production and increase in the relative weight of the spleen, with an increase in megakaryocytic nests.^20^ Additionally, the ethanolic extract of *H. sucuuba* bark promoted an increase in the synthesis and release of nitric oxide and tumor growth factor-alpha (TNF-α) and a reduction in transforming growth factor-beta (TGF β) by macrophages,^25^ suggesting that the gender *Himatanthus* exert immunomodulatory activity *in vivo*. On the other hand, EEHB administration promoted no change in the serum biochemical markers of functional activity, relative weight, and histological features of kidneys and liver. These data seem to suggest that the use of EEHB in the murine model of sarcoma 180 was safe.

Due to the EEHB-induced Caspase 3 immunohistochemical overexpression, the docking fitness scores of the bioactive conformations of the major phytochemical compounds and their specificity for caspase-3, the docking of plumeride and isoplumeride was performed at the active site of the caspase-3 enzyme to explore their binding modes as caspase-3 stimulators using the AutoDock Vina 4.^33^ The crystal structure of the caspase-3 protein (PDB: 3DEI protein data bank)^58^ was used as a good template for many validation sets of structure-based pharmacophore modeling of caspase-3 stimulators.^59^ The interactions of plumeride and isoplumeride showed optimal binding features,^60^ which is suggestive that both molecules might work as potential activators of caspase-3.^61^ Molecular docking results supported the immunohistochemical overexpression of caspase-3 active induced by EEHB observed in the current study and seems to suggest that this extract could be a potential anticancer drug candidate.

Pharmacokinetic tool predictions, i.e., absorption, distribution, metabolism, excretion, toxicity (ADMET) was performed using the online PreADMET software which comes with an advantageous space for drug discovery.^62^ *In silico* BBB tests are relevant tools to help predict the brain absorption of drug candidates from *in vivo* studies. We found that plumeride and isoplumeride showed deniability to permeability in the BBB, suggesting that the EEHB major chemical compounds present no potential activity on the central nervous system (CNS). The classification regarding permeability in Caco-2 cells according to pharmacokinetic studies is presented as follows: >70 nm/s: high permeability, 4–70 nm/s: medium permeability, >4 nm/s: low permeability. Plumeride and isoplumeride presented medium skin permeability, suggesting the possibility of use in topical formulations.^63^ As the CYP-450 enzyme complex has subfamilies responsible for the drug metabolism process, such as CYP3A4, CYP2C19, CYP2C9, CYP2D6 ^64^, the inhibition of CYP450 components suggests slow metabolism and excretion, which can lead to bioaccumulation in the body.^65^ On the other hand, the phytochemicals were substrate for CYP3A4 and CYP2D6, responsible for the drug metabolism process, suggesting that they may influence the metabolism and bioactivation of co-administered drugs.^64^ The model of Madin-Darby canine kidney (MDCK) was assessed to measure the rate of drug elimination, as this cell simulates an intestinal epithelial barrier and the property of renal clearance.^66^ As the higher the MDCK value presented, the greater the excretion capacity of this compound (Volpe, 2008), our data suggest low excretion via the MDCK pathway. Furthermore, the metabolism and excretion of many drugs also depend on PgP activity, a cell surface protein involved in Xenos efflux from the cell.^67^ Both the compounds are not substrates for PgP, suggesting that would not undergo efflux through this pathway. However, plumeride showed to be a PgP inhibitor, suggesting the possibility of reducing the efflux of other drugs, which are known to be recognized by PgP, which are occasionally co-administered with EEHB.^68^ Both the compounds in question proved to be very soluble, suggesting poor absorption potential due to the impossibility of crossing lipid-rich cell membranes. AMES Test demonstrated that only isoplumeride is mutagenic. Both compounds showed carcinogenic potential in mice, whereas in rats only isoplumeride was positive. These data, based on data from the NTP (National Toxicology Program) and the Food and Drug Administration – USA, suggest these substances can promote genetic or epigenetic changes that can lead to cancer.

## 5 Conclusions

Extract of stem bark of *Himatanthus bracteatus* contains higher iridoids compound and shows antitumoral activity, which involves a heterogeneous and complex mechanism related the induction of the apoptosis cascade. The data presented in our study suggest antitumor activity of EEHB in the experimental model of sarcoma 180, possibly resulting from increased apoptosis of tumor cells associated with caspase-3 overactivation. The prediction of ADMET properties indicates that the extract might be an interesting candidate for further drug development. However, further detailed studies are needed to elucidate the precise biochemical pathways related to the antitumor effects of the extract. Additional *in vitro* and *in vivo* analyses are required for the identification and isolation of the active molecules that are responsible for the cell phenotype alterations observed in our study. To our knowledge, our study reported here is the first work that brings to light evidence that the ethanolic extract of *H. bracteatus*, obtained through sequential extraction under high pressure conditions, can exhibit potential antitumor activity *in vivo*, without major repercussions on the immunohematological system of the animals (unlike the chemotherapy used as control).

## Author contributions

All authors have actively contributed to the conception and design of the study, data acquisition, analysis and interpretation, drafting the article and revising it critically for important intellectual content, and have approved the final version to be submitted.

## Data availability

Data will be made available from corresponding authors upon reasonable request.

## Consent to participate

Not applicable.

## Consent to publish

Not applicable.

## Ethics issues

This work raises ethics issues as part of the animal experimentation. Ethics approval was issued by the National Animal Experiment Control Council (CONCEA-Brazil) (approval nº 031115).

## Declaration of competing interest

The authors declare that they have no known competing financial interests or personal relationships that could have appeared to influence the work reported in this paper.
