# Supplementary material for: Himatanthus bracteatus stem bark ethanolic extract obtained by sequential pressurized liquid extraction: Chromatographic characterization and profiling of cytotoxic, antitumoral and immunopharmacological properties
Source: J Tradit Complement Med. 2024 Jun 12;15(3):319–29. doi: 10.1016/j.jtcme.2024.06.004 (PMC12143339; doi:10.1016/j.jtcme.2024.06.004)
Supplement: Multimedia component 1 [file mmc1.docx]

**Supplementary**

**Figure S1**. Chromatograms and mass spectra of the ethanol extract of *H. bracteatus* obtained using pressurized liquids.
